# Single cell labeling combined with electrophysiological identification

**DOI:** 10.1007/s12565-025-00882-x

**Published:** 2025-08-12

**Authors:** Takahiro Furuta

**Affiliations:** Dept. Systematic Anatomy and Neurobiology, Grad. Sch. Dentistry, Osaka University, Suita, 565-0871 Japan

**Keywords:** Cell labeling, Single unit recording, in vivo experiment

## Abstract

Gaining insight into the circuit architecture of neural tissue significantly aids in the investigation of nervous system mechanisms. Since neural networks are constituted by connections between axons and dendrites derived from neurons, morphological analysis of each neuron is useful to reconstruct circuit diagrams of neural circuits in detail. Here, I introduce a method where we can combine single unit recordings and visualization of neuronal morphology at a single neuron level. This technique reveals morphological characteristics of neurons which are electrophysiologically identified.

## Introduction

Nervous system contains enormous neurons that possess many long processes and constitute complicated networks. Since neuronal wiring structure is considered to be deeply related with the algorithm of information processing in neural networks, a lot of researchers investigate morphology of axons and dendrite to reveal architecture of neural networks. On the other hand, analyses for firing activities of neurons in the brain are crucial to discuss mechanisms of nervous systems. With the aim of studying morphological characteristics and firing properties at the individual neuron level, “juxtacellular labeling” technique was developed (Pinault 1996). In juxtacellular labeling, single unit recording is performed on a neuron, and then, small molecule neural tracer (neurobiotin) is injected into the recorded neuron to visualize the neuronal shape. This technique is so powerful that we are able to obtain both firing and morphology data for each identified neuron. However, a drawback of this method is that the tracer is easily metabolized within the cells, making it difficult to visualize even the distant part from the cell body. In the previous article (Furuta et al. 2011), we modified this method to visualize whole figure of labeled neurons by employing long-lasting neural tracer, biotinylated dextran amine (BDA). Here, I introduce juxtacellular labeling technique using BDA.

## Materials

### Animals

We mainly used male Wistar rats (250–350 g body weight) for this method. This animal experiment involving animal care, surgery, and sample preparation were approved by the Institutional Animal Care and Use Committees of Osaka University (Approval Nos. 300150 and R050160) and conducted in accordance with Fundamental Guidelines for Proper Conduct of Animal Experiments by the Science Council of Japan. All efforts were made to minimize animal suffering and the number of animals used.

### Biotinylated dextran amine (BDA)

Dextran, Biotin, 3000 MW, Lysine Fixable (BDA-3000, ThermoFIsher, Waltham, MA).

### Glass capillary

Borosilicate glass capillaries (1.5 mm outer diameter, 0.86 mm inner diameter, GC150F-10, Harvard Apparatus, Holliston, MA).

### Current clamp amplifier

IR-183A Intracellular Recording Amplifier (Cygnus Tech, Delaware, USA).

### Stereotaxic apparatus

SR-6R and SM-11 (Narishige, Tokyo, Japan).

### Manipulator

One-axis Motorized Stereotaxic Micromanipulator DMA-1650 (Narishige).

## Methods

### BDA solution

BDA powder was resolved in 0.5 M potassium acetate to a concentration of 10% just before use. We used 1–2 µl of BDA solution for each recording pipette. See the Note (N1) also.

### Glass pipette

Prepare sharp electrodes from glass capillaries using a puller equipped with a large heater. Make the electrode tips blunt (~ 1 µm diameter) by bumping the tip to something hard under a light microscope.

### Recording and labeling

**Fig. 1:**
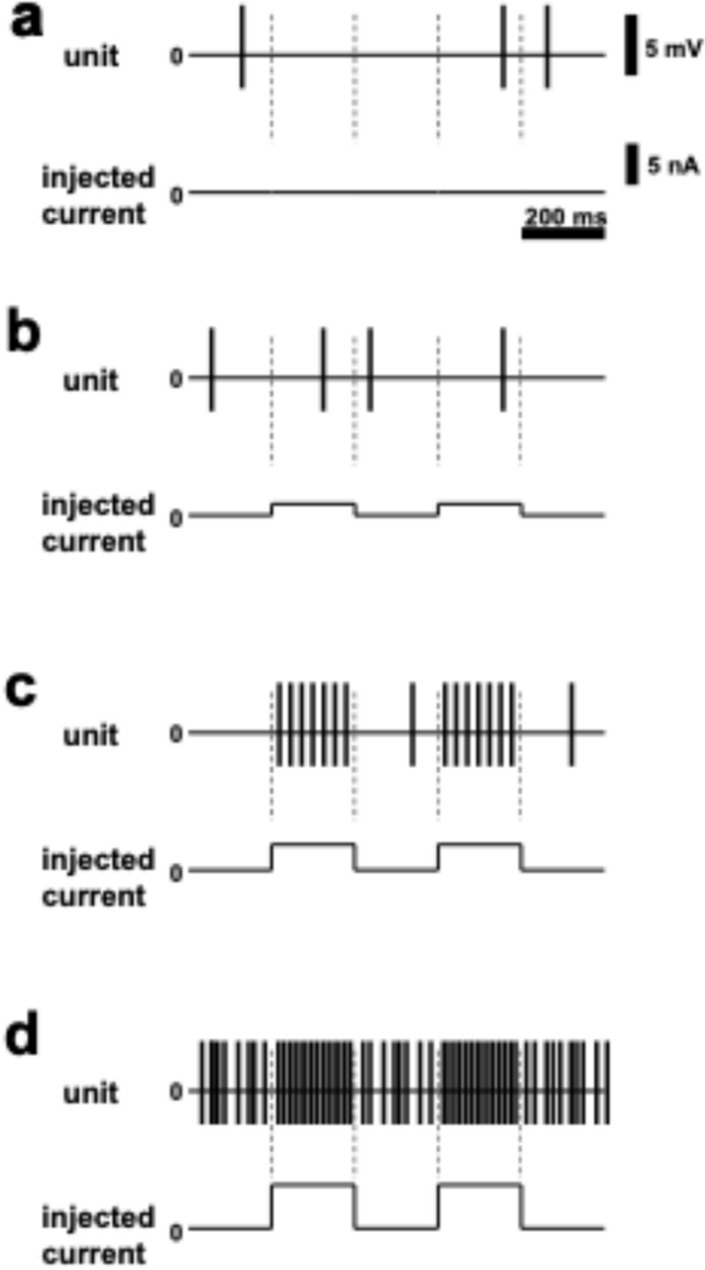
Schematic drawing explaining relationship between intensity of current pulses and numbers of evoked spikes: **a** condition before application of current pulses; **b** only a few evoked spikes are observed with small current pulses. The optimal situation of juxtacellular labeling is exhibited by **c**. Condition **d** indicates that the injected current is too strong

Experiments are performed under isoflurane (1% (v/v)) anesthesia. Placed the rat in a stereotaxic apparatus, breathed freely, and maintain the body temperature at 37 °C with a heating pad. After making a burr hole on the skull above the target brain structure, a small cut is made on the dura by a small syringe needle. Lower a glass pipette which is connected to a current clamp amplifier and contains BDA solution through the cut on the dura to place the pipette tip at the recording site. Move the pipette down slowly (around 1 µm/second) to obtain spike waves as single unite recording. During establishing the single unit recording, use spontaneous firing or evoked firing (e.g., sensory stimulation evoking, antidromic stimulation evoking, and optogenetical stimulation evoking). Record firing activity extracellularly. Before juxtacellular labeling, adjust the distance between the pipette tip and the cell body according to the size of spikes. We usually adjust the place of the pipette to have spikes of 3–5 mV amplitude, although the optimal size of spikes should be determined in each experiment set (also see Note N2). Apply positive current pulse injection (200 ms ON and 200 ms OFF) and increase gradually the intensity of the current (from 0 nA) until having modulated firing activities which are evoked by injected current pulse through the pipette (Fig. 1). Adjust the current intensity flexibly to prevent the firing rate from becoming too high or too low and keep the modulated firing activities for 10–15 min (also see Note N3). After completing the procedure, the pipette is retracted slowly (around 1 µm/second near the cell body), the skin is sutured, and the rat is administered analgesics and then returned to the animal facility.

### Fixation and sectioning

Following a survival period of 7–10 days (also see Note N4), the rat is sacrificed by transcardial perfusion. Administer deep anesthesia to the rat by an intraperitoneal injection of a mixture of medetomidine (0.3 mg/kg; Domitor, Zenoaq), midazolam (4 mg/kg; Dormicum, Astellas Pharma), and butorphanol (5 mg/kg; Vetorphale, Meiji Seika Pharma). Subsequently, perform transcardial perfusion with 200 ml of phosphate-buffered saline (5 mM PBS), followed by 300 ml of fixative solution (4% paraformaldehyde in 0.1 M phosphate buffer pH7.4). Upon completion of perfusion, carefully extract the brain from the skull and transfer it into a container filled with the same fixative solution. After an immersion fixation period of 1 h, replace the fixative with 30% (w/w) sucrose in PBS for cryoprotection. After cryoprotection of 20 h, cut the brain into sections of 30–50 µm thickness on a freezing microtome. The sections are collected with a brush and stored in 0.5 mM PBS at 4 °C.

### Histology

**Fig. 2:**
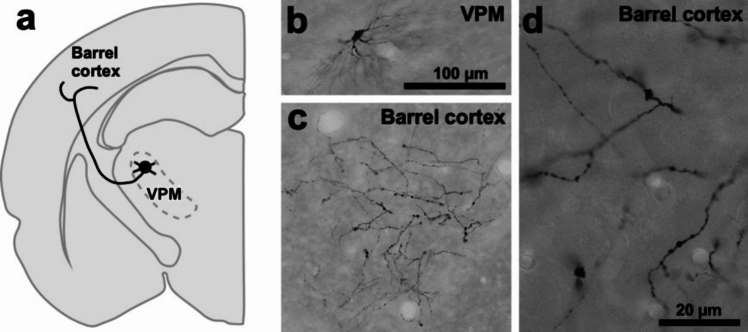
A representative example of juxtacellular labeling with BDA. **a** Schematic drawing showing locations of the labeled cell body and axons. **b** The cell body and dendrites of the labeled neuron were visualized in the ventral posterior medial nucleus (VPM) of the thalamus. **c** Labeled axons which were derived from the labeled thalamic relay neuron were found in the barrel area (barrel cortex) of the primary somatosensory cortex. **d** The labeled thalamocortical axon in the barrel cortex (**c**) are magnified

Wash the sections twice for 10 min in PBS. Incubate the sections in blocking solution, PBS containing 10% normal donkey serum and 0.3% Triton X-100 (also see Note N5), for 30 min. Sections are consecutively incubated for 2 h at room temperature with avidin-biotinylated peroxidase complex solution (ABC-Elite kit; Vector Laboratories, Burlingame, CA) in the same buffer with the blocking solution. Wash the sections twice in Tris–HCl buffer. Finally, the bound peroxidase is developed through a reaction with 0.02% (w/v) diaminobenzidine-4HCl (DAB) and 0.001% (v/v) hydrogen peroxide (H_2_O_2_) in 50 mM Tris–HCl buffer, pH 7.6. The sections were mounted onto gelatin-coated glass slides, air dried, dehydrated, defatted, and cover-slipped. A representative example of juxtacellular labeling in thalamocortical neurons is shown in Fig. 2. Thalamocortical projection axons which were derived from a single neuron were clearly visualized by BDA injection, because we can wait for 7–10 days, while neurobiotin (or biocytin) which is injected by the conventional juxtacellular labeling method fade in a few days.

Note:

N1. Prepared BDA solution might be stored at 4 °C for several days. We did not confirm whether the solution works after a long retention period (~ several weeks).

N2. When a recording pipette is placed at the optimal position, artificially evoked firing activity should be steadily driven by injected current pulses. The spike size during the optimal pipette placement should be memorized to reproduce the steady juxtacellular injection, because spike size depends on the positional relationship between the recorded cells and recording pipettes.

N3. When the distance between the recording pipette and the recorded (injected) cell is appropriate, spikes should be evoked by moderate pulses which are smaller than 10 nA. If the applied pulses are too strong, the cell might be killed. Intensity of pulses should be continuously adjusted (see Fig. 1).

N4. It is also important to optimize the survival time for each research project. First of all, BDA stay in the labeled cell after a long survival time (10 days and more), while neurobiotin, which has been used for conventional juxtacellular labeling, is almost diminishing 24 h after the injection. Experimenters might wait for a long survival time to have the tracer (BDA) reach the ends of long projecting axons. In our experience, it takes about 7 days to visualize thalamocortical axons. Because optimal survival time depends on the neuron type of interest of each project, the survival time should be determined by each researcher based on the standard of 7–10 days.

N5. This method is usable combined with electron microscopy. When a researcher wants to subject juxtacellular labeled cells to electron microscopic analyses, detergents, such as Triton X-100, should not be used, because membrane structures are damaged by detergents at an electron microscopic level. Staining of axons with solutions which does not contain any detergent is inferior to that with solutions containing detergent, but it should be still observable.
